# Downregulation of C-Terminal Tensin-Like Protein (CTEN) Suppresses Prostate Cell Proliferation and Contributes to Acinar Morphogenesis

**DOI:** 10.3390/ijms19103190

**Published:** 2018-10-16

**Authors:** Wei-Ming Wu, Yi-Chun Liao

**Affiliations:** Department of Biochemical Science and Technology, National Taiwan University, No. 1, Sec. 4, Roosevelt Road, Taipei 10617, Taiwan; f99b22054@ntu.edu.tw

**Keywords:** C-terminal tensin-like protein (CTEN), prostate, proliferation, acinar morphogenesis, focal adhesion kinase (FAK)

## Abstract

C-terminal tensin-like protein (CTEN) is a member of tensin family, which is crucial for the assembly of cell-matrix adhesome. Unlike other tensins, CTEN is selectively expressed only in a few tissues such as the prostate. However, the biological relevance of CTEN in normal prostate is poorly understood. In this study, we revealed that CTEN is selectively expressed in the prostate epithelial cells and enriched in the basal compartment. Knockdown of CTEN in RWPE-1 cells suppresses cell proliferation and results in G1/S cell cycle arrest as well as the accumulation of cyclin-dependent kinase (CDK) inhibitors, p21 and p27. Moreover, the expression of CTEN is decreased during acinar morphogenesis using Matrigel-based three-dimensional (3D) culture. In the course of acinar formation, induction of CTEN reactivates focal adhesion kinase (FAK) Y^397^ phosphorylation and disrupts the acini structure. This study, to our knowledge, is the first report demonstrating that downregulation of CTEN is required for luminal differentiation and acinar formation.

## 1. Introduction

Prostate is a compound tubuloalveolar gland encircling the beginning of urethra below the neck of bladder. It originates from urogenital sinus and develops into mature prostate through four stages, including sexual dimorphism, prostate budding, branching morphogenesis, and epithelial differentiation [1]. In the last stage of prostate organogenesis, urogenital epithelial progenitor differentiates into several cell types, and then glandular lumen is formed as well as the prostate epithelium functionally matures. A normal human prostate is composed of epithelium, neuroendocrine cells, fibroblasts, muscle cells, and endothelial cells, mainly based on their morphologies. Adult prostatic epithelium is majorly composed of basal, luminal and intermediate cells. The flattened basal cells directly contact basement and form a continuous layer surrounding luminal cells. They are characterized by integrin α2β1, α6β4, cytokeratin 5 (CK5) and ΔNp63. The tall columnar luminal cells differentiate from progenitor cells in basal compartment and then move toward gland lumen. They are identified by cytokeratin 8 (CK8), cytokeratin 18 (CK18) and androgen receptors (ARs). The intermediate cells express both basal and luminal cell markers as well as prostate stem cell antigen (PSCA). It has been suggested that irregular control of epithelial differentiation is associated with prostate cancer and benign prostatic hyperplasia (BPH) [2,3].

CTEN was identified as a member of tensin family, which is mainly localized at focal adhesions [4]. Tensin family proteins all share highly conserved Src homology 2 (SH2) and phosphotyrosine-binding (PTB) domains at the C-terminus. However, CTEN has some unique features. First, CTEN homologies are found only in mammals, suggesting that it appears to be a newly evolved gene [5]. Secondly, human CTEN is a 76.8 kDa protein, about half of other tensins, and lacks the conserved N-terminal actin-binding domain found in other tensins. In addition, while tensin 1, 2 and 3 represent broad expression patterns in normal tissues, CTEN is enriched in prostate and placenta [4,5]. Moreover, *CTEN* promoter is more active in prostate than many nonprostatic cells or tissues [5]. These studies imply that CTEN is probably linked to the development of mammalian features. Previously, we have shown that CTEN mediates prostate cell adhesion and is transcriptionally regulated by ΔNp63α [6]. ΔNp63α is the predominant isoform in basal compartment of prostate epithelium and loss of p63 in male mice results in the absence of prostate [7]. By using renal grafting, prostatic tissue in p63^−/−^ mice developed and displayed incomplete lineage specification of prostate epithelium [8,9]. Moreover, CTEN is a Nkx3.1 target gene and downregulated by Nkx3.1 during prostate differentiation [10]. Nkx3.1 is expressed in epithelium during prostate organogenesis and its expression in adults is predominant in prostatic luminal cells [1,10,11,12,13,14]. It is suggested that Nkx3.1 is responsible for luminal differentiation and regular lumen space [10,11,14].

Based on the above-mentioned findings, we speculate that CTEN might act as a key factor in the development of prostate epithelium. To date, the distribution of CTEN in prostate has not been clarified and the functional role of CTEN in prostate is poorly investigated. In the present study, we first analyzed the CTEN expression profile in prostate. We also elucidated the role of CTEN in prostatic epithelial cell proliferation. Moreover, by using a 3D culture system, we demonstrated that CTEN is downregulated in cells undergoing acinar morphogenesis. Our results unravel a novel role of CTEN contributing to acinar differentiation by modulating the phosphorylation of focal adhesion kinase (FAK).

## 2. Results

### 2.1. CTEN Is Highly Expressed in Prostate Basal Epithelial Cells

**Figure 1:**
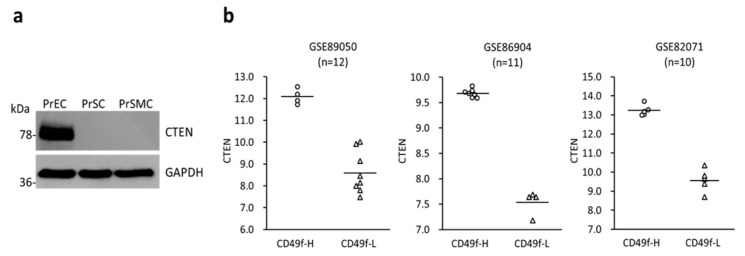
C-terminal tensin-like protein (CTEN) is enriched in the basal type of prostatic epithelial cells. (**a**) The levels of CTEN protein in the prostate epithelial (PrEC), stromal (PrSC) and smooth muscle (PrSMC) cells were examined by Western analyses using the indicated antibodies. Glyceraldehyde 3-phosphate dehydrogenase (GAPDH) was used as a loading control. (**b**) Gene expression data from the indicated datasets was divided to two groups based on the levels of a prostate basal cell marker, CD49f. The levels of CTEN transcripts in the high-CD49f (CD49f-H, ◯) and low-CD49f (CD49f-L, △) expression group were presented as a dot plot. The black line indicated the average of CTEN expression.

The distribution and location of CTEN protein in normal cells are of particular importance in its biological activities. Previous studies have demonstrated that CTEN is highly expressed in prostate [4,5] but the expression pattern in various types of prostate cells has not been determined. To clarify the cell-type-specific expression of CTEN, we first examined the levels of CTEN protein in primary epithelial, stromal and smooth muscle cells isolated from human prostate by Western analyses. The result showed that CTEN protein is highly abundant in the prostate epithelial cells but nearly undetectable in the prostate stromal and smooth muscle cells (Figure 1a). Next, we further investigated the distribution of CTEN in the prostate epithelium by the analyses of publicly available online databases. Three datasets, including GSE89050, GSE86904 and GSE82071, were obtained from Gene Expression Omnibus (GEO) and their gene expression profiles were analyzed by microarray (GSE89050 and GSE86904) or RNA-sequencing (GSE82071). In these datasets, benign human prostate specimen was dissociated into single cell and fluorescence-activated cell sorting was performed to separate basal epithelial cells from luminal ones as described in Materials and Methods. We interrogated the expression of CTEN in prostate basal and luminal epithelial cells, which were discriminated based on the levels of CD49f (aka integrin α6), a prostate basal cell marker [15]. In all the three datasets, CTEN mRNA transcripts are greatly increased in the subpopulation detected with high levels of CD49f (CD49f-H) compared to that detected with low levels of CD49f (CD49f-L) (Figure 1b). It indicates that CTEN is predominantly expressed in the prostatic basal epithelial cells but decreased in the luminal subtypes.

### 2.2. Depletion of CTEN Attenuates Prostate Cell Proliferation

**Figure 2:**
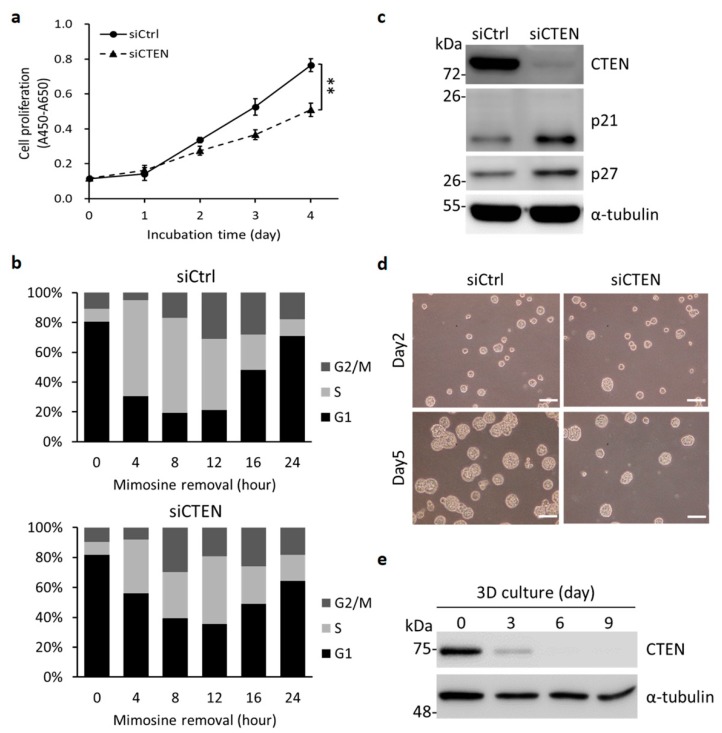
Knockdown of CTEN suppresses cell proliferation and downregulation of CTEN occurs during cell differentiation. (**a**) RWPE-1 cells were transfected with CTEN-specific siRNA (siCTEN) or nontargeting siRNA (siCtrl). Cell proliferation was evaluated by WST-1 based assay as described in Materials and Methods. Data were expressed as mean ± standard deviation (SD) of three independent experiments analyzed in triplicate (Student’s *t* test; ** *p* < 0.01). (**b**) Cell cycle phase distribution (G1, S and G2/M) was determined by propidium iodide staining and flow cytometry. (**c**) Two days after transfection with the indicated siRNA, the protein levels were examined by Western analyses using the indicated antibodies. α-tubulin was used as a loading control. (**d**) One day after transfection with the indicated siRNA, RWPE-1 cells were subcultured in 3D cultivation system (day 0). The images of acini structure were acquired by a phase contrast microscope at the indicated time and the representative images were shown. The experiments were repeated in three independent cultures and similar results were obtained. Scale bar: 100 μm. (**e**) RWPE-1 cells grown in 3D culture were collected at the indicated time and the protein levels were examined by Western analyses using the indicated antibodies. α-tubulin was used as a loading control.

The epithelial cell-restricted and basal cell-enriched expression of CTEN in prostate led us to speculate that CTEN might have a role in maintaining the basal compartment. To test this hypothesis, we performed siRNA-mediated gene silencing of CTEN in RWPE-1 cells. RWPE-1 is a nonmalignant human epithelial cell line derived from a histologically normal adult human prostate and is used as a benign prostatic epithelial cell model to study the regulation of growth and differentiation [16,17]. In addition to suppression of cell adhesion [6], knockdown of CTEN resulted in reduced cell proliferation (Figure 2a). To test whether silencing of CTEN could impede cell cycle progression, RWPE-1 cells were transfected with nontargeting control (siCtrl) or CTEN-specific siRNA (siCTEN) followed by being stained with propidium iodide (PI) for cell cycle analyses using flow cytometry technique. After transfection with siRNA, the cells were treated by thymidine/L-mimosine and then the chemical blockade was removed for releasing cells to synchronize at late G1 phase. Four hours after releasing from L-mimosine blockade, the ratio of S phase subpopulation in the siCtrl group increased to 53% whereas only 26% in the siCTEN group (Figure 2b). Cell cycle phase distribution suggests that CTEN deficiency causes a hindrance to G1/S phase transition. In addition, knockdown of CTEN enhanced the accumulation of cyclin-dependent kinase (CDK) inhibitor p21 and p27 (Figure 2c). These findings suggest that depletion of CTEN attenuates prostate cell proliferation by the cell cycle arrest.

Moreover, to examine the possibility that CTEN is involved in prostate gland differentiation, a 3D in vitro model of human prostate acinar formation was applied using the RWPE-1 cell line, which is reported to form consistent structures representative of normal adult human prostate glands [16,18,19]. The RWPE-1 cells transfected with control siRNA formed round spheroids in Matrigel after 5 days of incubation (Figure 2d). On the other hand, the majority of the CTEN-depleted cells developed into phenotypically normal but formed fewer spherical structures compared to control (Figure 2d), indicating that loss of CTEN suppresses proliferation and leads to growth-arrested acinar formation. Interestingly, when we monitored the levels of CTEN in the course of acinar formation, we found that CTEN was also significantly reduced in the control acini, which were not transfected with siCTEN, after 3 days seeding in Matrigel (Figure 2e) It suggested that downregulation of CTEN occurs during acini morphogenesis and is associated with cell differentiation in the 3D culture of RWPE-1.

### 2.3. Downregulation of CTEN Is Required for Normal Acinar Formation

**Figure 3:**
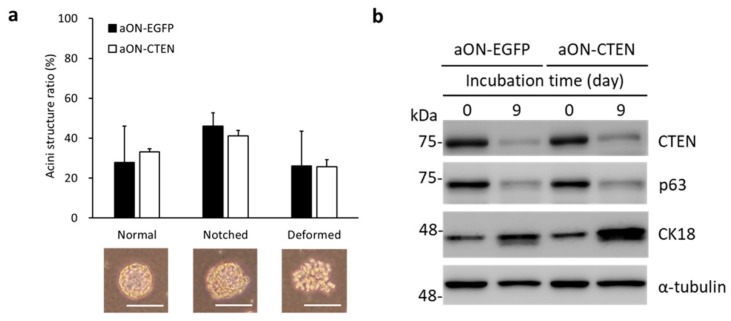
Inducible EGFP-expressing and CTEN-expressing RWPE-1 cells perform similar phenotypes in the absence of doxycycline in 3D culture. Inducible EGFP-expressing (aON-EGFP) or CTEN-expressing (aON-CTEN) RWPE-1 cells were incubated in 3D culture system for 9 days without doxycycline induction. (**a**) The appearance of acinar structures was observed and classified to three types (normal, notched and deformed). The numbers of the three-type acini in each group were counted and presented as a percentage of the whole (upper panel). Data were expressed as mean ± SD of three independent experiments. More than 50 acini were evaluated within each sample (Student’s *t* test; no significant difference between aON-EGFP and aON-CTEN in each phenotype). The representative images of acinar structure were also shown in the lower panel. Scale bar: 100 μm. (**b**) aON-EGFP and aON-CTEN RWPE-1 cells grown in 3D culture were collected at the indicated time and the protein levels were examined by Western analyses using the indicated antibodies. α-tubulin was used as a loading control.

Because CTEN is downregulated in the cells undergoing acinar morphogenesis, the inducible gene expression system was applied to study how CTEN affects the differentiation of RWPE-1 cells in 3D culture. Therefore, the tetracycline-inducible vector containing EGFP (aON-EGFP, as a control) or CTEN (aON-CTEN) was constructed and introduced into RWPE-1 by lentivirus infection. The stable cell pools were selected by puromycin and the optimal concentration of doxycycline (dox) inducer was determined by performing dose-response experiments. In the following morphogenesis assay, acini structures were classified into three groups based on their phenotypes, including normal (spheroid), notched (slightly disrupted spheroid) and deformed (disorganized cell mass) structure (Figure 3a, lower panel) [20]. In the absence of dox, both cell pools, aON-EGFP and aON-CTEN, developed similarly in 3D culture after 9 days, with around 30% of normal acini, 40% of notched spheres and 30% of deformed structures (Figure 3a). Furthermore, the differentiation markers were examined by Western analyses. After 9 days in 3D-Matrigel cultures, evaluation of the marker expression in both cell pools demonstrated a decrease of the basal cell marker p63 and an induction of the luminal cell marker CK18 (Figure 3b). It indicates that the progenitor properties of these two selected pools are similar and the 3D-culture system we used allows the expression of normal prostatic differentiation markers. Notably, CTEN is markedly downregulated after 9 days in culture (Figure 3b).

**Figure 4:**
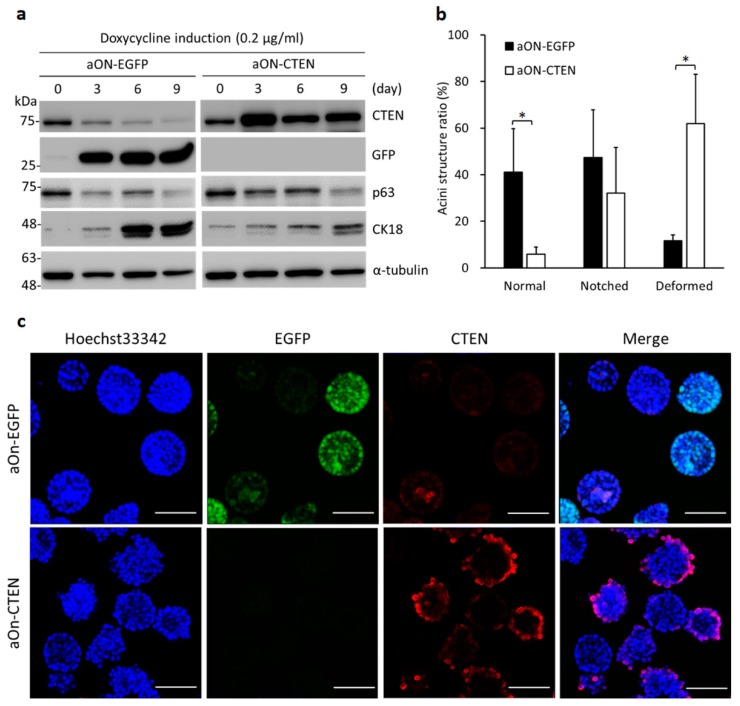
Forced CTEN expression disrupts acinar morphogenesis. Inducible EGFP-expressing (aON-EGFP) or CTEN-expressing (aON-CTEN) RWPE-1 cells were incubated in 3D culture system for 9 days in the presence of doxycycline (0.2 μg/mL). (**a**) Cells were collected at the indicated time and the protein levels were examined by Western analyses using the indicated antibodies. α-tubulin was used as a loading control. (**b**) The numbers of the three-type acini in each group were counted at Day 9 and presented as a percentage of the whole. Data were expressed as mean ± SD of three independent experiments. More than 50 acini were evaluated within each sample (Student’s *t* test; * *p* < 0.05). (**c**) The acinar structure was observed after 9 days by confocal microscopy. The fluorescence of ectopic EGFP was directly excited with 488-nm laser. The CTEN was detected by immunofluorescence staining with the antibody against CTEN followed by Alex flour 555 conjugated secondary antibody. Nuclear staining was carried out using Hoechst33342. Scale bar: 100 μm.

We next evaluated the role of CTEN during differentiation by acinar morphogenesis assay with dox induction. The cells from aON-EGFP or aON-CTEN pool were seeded in Matrigel and the expression EGFP or CTEN was induced respectively by dox treatment every other day during the assay. The cells were collected from 3D cultures at the indicated time point and analyzed by Western analyses. In aON-EGFP control, p63 (the basal cell marker) was reduced whereas CK18 (the luminal cell marker) was profoundly increased (Figure 4a), indicating that the cells undergo proper differentiation. Meanwhile, the levels of CTEN in aON-EGFP were downregulated during 9-day cultivation (Figure 4a). However, when CTEN was induced by dox in aON-CTEN 3D culture, the levels of p63 reduced with a slower rate and the increase in the levels of CK18 was significantly suppressed compared with the control (aON-EGFP) (Figure 4a). It suggests that the re-expression of CTEN might disrupt differentiation. In addition, more than 40% of acini formed in aON-EGFP cultures exhibited normal structures whereas less than 6% acini displayed normal phenotype in aON-CTEN cultures (Figure 4b). Re-expression of CTEN during acini morphogenesis significantly increased the number of deformed acini (Figure 4b). We also examined the acinar structure by immunofluorescence staining and confocal microscopy. After 9 days of incubation in the presence of dox, most of the acini formed in aON-EGFP cultures were spherical and the lumen formation was observed (Figure 4c). In contrast, most part of cells in aON-CTEN cultures formed solid cell mass (Figure 4c). Based on these findings, it is evident that keeping up CTEN expression disturbs acinar formation and luminal differentiation.

### 2.4. CTEN Interacts with Integrin β1 and Increases the Activity of FAK and RhoA

**Figure 5:**
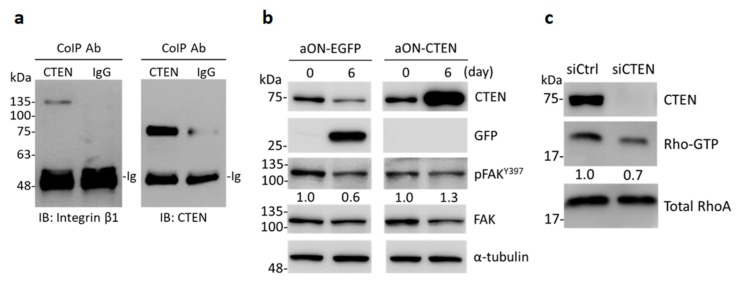
CTEN acts as a binding partner of integrin β1 and increases the activity of FAK and RhoA. (**a**) Whole cell lysate of RWPE-1 were coimmunoprecipitated (CoIP) with anti-CTEN antibody or normal rabbit IgG. The precipitates were examined by immunoblotting (IB) with anti-integrin β1 and anti-CTEN antibodies. (**b**) Inducible EGFP-expressing (aON-EGFP) or CTEN-expressing (aON-CTEN) RWPE-1 cells were incubated in 3D culture system for 6 days in the presence of doxycycline (0.2 μg/mL). The cells were collected at the indicated time and the protein levels were examined by Western analyses using the indicated antibodies. α-tubulin was used as a loading control. The densities of the protein bands from the Western blot were quantified. The levels of the phosphorylated FAK Y397 (pFAK^Y397^) was normalized to those of the total FAK. The ratio of pFAKY397/FAK in each group was presented. (**c**) RWPE-1 cells were transfected with the indicated siRNA. The GTP-RhoA in RWPE-1 lysates was purified using GTP-RhoA pull-down assays as described in Materials and Methods and examined by Western blot analyses with an anti-RhoA antibody. The protein levels in whole cell lysates were also examined by Western analyses using the indicated antibodies. Total RhoA was used as loading control. The densities of the protein bands from the Western blot were quantified. The levels of Rho-GTP was normalized to those of total RhoA. The ratio of Rho-GTP/total RhoA was presented.

It has been reported that CTEN interacts with integrin β1 and abolishing integrin β1 suppresses acinar morphogenesis [18,21,22]. Because RWPE-1 cells predominantly express α6β1 integrin receptor [18], we first examined the interaction between CTEN and integrin β1 by coimmunoprecipitation assay. Consistent with the previous findings [21], CTEN binds to integrin β1 as well in RWPE-1 cells, suggesting that CTEN might activate integrin β1 signaling through the interaction (Figure 5a). One of the important factors in integrin β1 signaling is focal adhesion kinase (FAK) and CTEN was previously found to facilitate the phosphorylation of FAK Y^397^ [23]. Therefore, we hypothesized that exogenous expression of CTEN in RWPE-1 during acinar formation would increase the levels of Y^397^-phosphorylated FAK (pY^397^-FAK) and in turn disrupt the acini structures. To test this, the expression of EGFP and CTEN were induced in the aON-EGFP and aON-CTEN cells, respectively, by dox treatment in 3D cultures. Cells were collected from Matrigel at Day 6 and the amounts of pY^397^-FAK and total FAK were determined by Western analyses (Figure 5b). During the 6-day cultivation, the relative pY^397^-FAK levels in the control (aON-EGFP) were decreased by a 0.6-fold reduction. Nonetheless, induced expression of CTEN in aON-CTEN cultures led to a 1.3-fold increase of pY^397^-FAK levels (Figure 5b). This result suggests that the phosphorylation of FAK Y^397^ is suppressed during acinar morphogenesis. Overexpression of CTEN causes higher levels of pY^397^-FAK and simultaneously disrupts organization of acini structures.

FAK plays critical roles in integrin-mediated signal transductions. To further determine which downstream signaling pathway is activated when CTEN is overexpressed, we next examined whether MAPK/ERK pathway participates in this regulation axis. Nonetheless, the levels of phosphorylated ERKs remain unchanged when CTEN is induced by dox. On the other hand, RhoA GTPase, which is also triggered by FAK, has been shown to regulate cell morphology and architecture in 3D cultures [24]. Disruption of acinar formation is associated with increased RhoA activation [25]. Therefore, we then analyzed the effect of CTEN on RhoA activation. RhoA activity was assessed using GTP-RhoA pull-down assays, which require at least 400 μg of protein. Cell lysate collected from 3D culture is not enough for this assay, so it was performed using RWPE-1 cells grown in monolayer cultures. RWPE-1 cells were transfected with non-targeting control (siCtrl) or CTEN-specific siRNA (siCTEN) and the active RhoA (GTP-RhoA) was pulled down after transfection. siCTEN totally abolished the expression of CTEN and the level of GTP-RhoA was reduced in samples transfected with siCTEN, demonstrating that RhoA is more activated in the presence of CTEN (Figure 5c).

## 3. Discussion

To date, CTEN-related research is majorly focused on cancer biology, the functional role of CTEN in normal prostate, where it is highly expressed, has not been well documented yet. We have previously demonstrated that ΔNp63α transcriptionally regulates CTEN in prostate epithelial cells, which in turn mediates cell adhesion [6]. ΔNp63α plays a crucial role in the regulation of epithelial integrity and prevents cells from anoikis [26,27,28]. Robust cellular adhesome is required for cell survival and CTEN therefore might act as a regulator of cell proliferation. Some studies have suggested that CTEN plays a positive role for cell proliferation in keratinocyte, lung and stomach cancer cells [23,29,30]. In this present study, we report for the first time that downregulation of CTEN suppresses prostate cell proliferation through the regulation of the cell cycle G1/S transition and such an arrest is associated with p21/p27 accumulations (Figure 2). The elevated levels in p21 and p27 were also observed in another nonmalignant prostate epithelial cell line, PZ-HPV-7, after the transfection with CTEN specific siRNA. The p21 and p27 belong to the CIP/KIP family of CDK inhibitor proteins, which inhibit the activities of cyclin D- and E-dependent kinases and controls the cell cycle progression at G1 checkpoint [31,32,33]. Our data suggest that the depletion of CTEN in prostate epithelial cells results in p21/p27 induction, which subsequently causes the inhibition of CDK/cyclin complexes, G1/S arrest and the suppression of cell proliferation (Figure 2).

Furthermore, our results unravel a novel role of CTEN contributing to acinar differentiation (Figure 4). We also found that CTEN is more abundant in basal cells than in luminal cells (Figure 1). During lumen formation of prostate epithelium, epithelial progenitor cells differentiate and the ratio of mitotic cells is decreasing [34]. In adult prostate, most highly proliferating cells are found in epithelial basal compartment [35,36,37]. At the initial stage of acinar formation, loss of CTEN by siRNA leads to growth-arrested acinar formation (Figure 2a,d), suggesting the existence of CTEN is required for supporting the proliferation of prostate basal cells. However, the subsequent acinar formation in the CTEN-knockdown cells are phenotypically normal (Figure 2d). This might be due to the fact that CTEN is also significantly downregulated in control RWPE-1 acini 3 days after seeding in Matrigel (Figure 2e). As a result, CTEN-knockdown cells form normal but fewer amount of spherical structures compared to the control (Figure 2d). On the other hand, because CTEN is regulated by ΔNp63α [6], the reduction of CTEN during acini morphogenesis might be due to the downregulation of the basal marker p63. In addition, CTEN might be also governed by other pioneer transcription factors such as Nkx3.1 [10] and executes specific events required for organization of acinar structure.

It has been reported that CTEN interacts with integrin β1 and the function-blocking of integrin β1 in prostate and mammary epithelial cells inhibits the formation of acinar structures [18,21,22]. However, elevated expression of integrin β1 in mammary epithelial cells still leads to aberrant acini structure [25]. These findings suggest that the appropriate regulation of integrin β1 signaling is essential to acinar morphogenesis. In our study, we demonstrated that CTEN interacts with integrin β1 in RWPE1 cell (Figure 5a). In addition to a decrease in CTEN (Figures 2e and 4a), the levels of pY^397^-FAK are reduced in the cells undergoing luminal differentiation (Figure 5b). FAK is one of the important factors in integrin αβ1 signaling. When CTEN is induced during acinar formation, overexpression of CTEN not only disrupts acini structure but also reactivates FAK phosphorylation (Figure 4 and Figure 5b). Previous studies have also revealed that the phosphorylation of FAK at Y^397^ residue impairs acinar morphogenesis [25,38]. It suggests that CTEN mediates normal acinar morphogenesis through modulating the activation of FAK. Disruption of acinar formation is associated with increased RhoA activation, which can be triggered by FAK [25]. The level of active RhoA was higher in the presence of CTEN (Figure 5c). Taken together, our results suggest that the disruption of acinar formation might be caused by FAK/RhoA activation due to the overexpression of CTEN.

3D culture system using the immortalized prostate epithelial cell line RWPE-1 was used to investigate the acinar morphogenesis in our study. Although rodent models, the most accessible and acceptable in vivo experimental platform, are widely used for the study of prostate organogenesis; however, many morphological differences in the prostate exist among mammals. The rodent prostate consists of three distinctive lobes but the human prostate is a compact structure containing three zones [39,40]. In addition, in human prostate the architecture of basal cell layer and the ratio of basal to luminal cells are different from other animal species [41]. Therefore, the use of a human based experiment model to study prostate development is indispensable. Some strategies, such as ex vivo organoid cultures and human primary epithelial cell model with conditioned medium, have been proposed [42,43,44,45,46] and these systems are beneficial to dissect the molecular detail determining cell fate. The RWPE-1 cell line used in our study retains normal functional abilities and is widely used for studying acinar morphogenesis of the normal human prostate [16,17,18,19,20,47]. Moreover, RWPE-1 possesses potential to differentiate toward luminal subtypes [16,48]. Incubation of RWPE-1 on Matrigel with complete medium leads to formation of acinar, cord-like and deformed structures. In order to focus on acinar morphogenesis, the related event during prostate organogenesis, we used the 3D culture system previously proposed by Tyson et al., which allows RWPE-1 to form more acinar and less cord-like or deformed structures [19]. We further demonstrated that the 3D-culture model allows RWPE-1 cells to differentiate toward luminal cells by the examination of CK18 (luminal marker) and p63 (basal marker) (Figure 3b). Overall, the RWPE-1 3D culture system is suitable as a physiologically relevant system to study acinar morphogenesis as well as differentiation and is complement to other sophisticated models.

Several lines of evidence have demonstrated that CTEN is upregulated in many types of cancer and participates in cell motility, apoptosis, epidermal growth factor signaling, and tumorigenicity [49]. However, in prostate cancers and BPH, CTEN expression is frequently reduced [4,5,6,50]. CTEN protein levels are inversely correlated with pathological Gleason scores of prostate cancer patients [50]. These findings imply that the role of CTEN is diverse and depends on cellular context. Most prostate cancers are characterized by luminal phenotypes [51]. Our work has demonstrated that CTEN is abundant in basal cells and downregulation of CTEN occurs in luminal differentiation (Figure 1b, Figure 2e and Figure 4), which might further contribute to cell transformation. Research about cellular origin of prostate cancers is controversial. Some studies proposed that prostate cancer can arise from luminal cells and some suggested from basal cells [13,52,53,54,55,56,57]. However, these findings reveal that luminal differentiation is prior to full transformation. Even though the downregulation of CTEN might be a background change during prostate tumorigenesis, we cannot exclude the causative role of CTEN loss in prostate cancers. We have previously revealed the negative relationship between ΔNp63α-CTEN regulatory axis and prostate cancer progression [6]. In addition, forced expression of CTEN in prostate cancer cell line has been demonstrated to elevate the cell sensitivity to Paclitaxel [50]. Therefore, CTEN might act as a potential tumor suppressor in prostate cancers. Nevertheless, the function of CTEN in prostate cancers still remains to be explored. The programs activated during prostate organogenesis would also contribute to prostate tumorigenesis [58,59]. Some proteins with cell-subtype specific expression, such as p63, Nkx3.1 and Dickkopf-3, have been demonstrated to play important roles in the development of prostate epithelium [8,10,14,60,61]. Our work highlights the distribution and the expression regulation of CTEN in prostate epithelium, which is physiologically relevant for the development and homeostasis of prostate epithelium. Investigation of the normal tissue development would provide a sound basis for the cancer research.

## 4. Materials and Methods

### 4.1. Cell Culture

Human primary prostate epithelial (PrEC), stromal (PrSC), and smooth muscle (PrSMC) cells were obtained from Lonza (Allendale, NJ, USA). Lonza’s PrECs are isolated from the glandular epithelium (parenchymal portion) of the prostate. PrSCs are a mixed population of cells isolated from the stroma of the prostate and the majority of the cells are fibroblastic in origin. PrSMCs are isolated from the stroma and the vasculature of the prostate. They were maintained using their respective medium kits recommended by Lonza. RWPE-1 cells (American Type Culture Collection, ATCC, Manassas, VA, USA) were cultured in keratinocyte serum free medium (K-SFM) (Invitrogen, Carlsbad, CA, USA) supplemented with 0.05 mg/mL bovine pituitary extract, 5 ng/mL human recombinant epidermal growth factor and 1% penicillin/streptomycin. All cells were maintained at 37 °C in a humidified atmosphere containing 5% CO_2_.

### 4.2. Bioinformatics Analyses

The gene expression datasets, including GSE89050, GSE86904 and GSE82071, were downloaded from Gene Expression Omnibus (GEO). The criteria of purification in these three datasets are slightly different. In GSE89050 dataset, CD45−/EpCAM+/CD49fHi is for basal cells and CD45−/EpCAM+/CD49fLo is for luminal cells. In GSE86904 dataset, EpCAM+/CD44−/CD49fHi is for basal cells and EpCAM+/CD44+/CD49fLo is for luminal cells. In GSE82071 dataset, Trop2+/CD49fHi is for basal cells and Trop2+/CD49fLo is for luminal cells. In this study, basal and luminal types of benign prostate epithelial samples from the datasets were analyzed. Quantile normalization of data sets was performed on NetworkAnalyst (available online: http://www.networkanalyst.ca/faces/home.xhtml). The *CTEN* expression signal was acquired to generate the dot plot.

### 4.3. RNA Interference

Stealth RNAi siRNAs against CTEN (Invitrogen) were used in the knockdown experiment. Pre-designed negative control siRNA (Sigma-Aldrich, St. Louis, MO, USA) was used as a control. Cells were transfected with siRNA (50 pmol) by using Lipofectamine 2000 (Invitrogen) according to manufacturer’s instructions.

### 4.4. Cell Proliferation Assay

Knockdown of CTEN gene expression was performed by siRNA-mediated gene silencing. Twenty-four hours after transfection, RWPE-1 cells were suspended in complete growth medium and 100 μL of cell suspension (4000 cells) was dispensed into each well of 96-well culture plates. At the indicated time, culture medium was replaced with 100 μL of 5% (*v*/*v*) WST-1 (Roche, Indianapolis, IN, USA) solution (diluted with complete growth medium) and cells were incubated at 37 °C for 3 h. Cell proliferation index was determined by subtracting absorbance of 650 nm from absorbance of 450 nm.

### 4.5. Cell Cycle Analyses

Knockdown of CTEN gene expression was performed by siRNA-mediated gene silencing. Six hours after transfection cells were treated with 2 mM thymidine (Sigma-Aldrich) for 12 h. For allowing cells exit from S phase, the thymidine medium was replaced with complete growth medium. Next, cells were treated with 400 μM L-mimosine (Sigma-Aldrich) for 14 h. In order to release cells from chemical blockade, the L-mimosine medium was replaced with complete growth medium (time 0). At the indicated time, cells were suspended in chilled 70% ethanol and fixed at −20 °C overnight. For analyzing cell cycle phase distribution by DNA content, ethanol was removed and PI/RNase staining solution (BD Pharmingen, San Diego, CA, USA) was added to suspend cells. Cells were incubated at room temperature for 15 min and then analyzed by flow cytometry BD FACSCanto II.

### 4.6. Constructions of Lentiviral Expression System

Flag-tagged CTEN and enhanced green fluorescent protein (EGFP) inserts were amplified by PCR from Flag-CTEN [62] and EGFP-CTEN [63] plasmids, respectively, and subcloned in frame into pAS4.1W-Ppuro-aOn (National RNAi Core Facility, Taipei, Taiwan), an all-in-one tet-inducible lentiviral transfer vector. Lentiviral particles were generated in 293T with pMD.G, pCMVΔ8.91 (National RNAi Core Facility) and lentiviral transfer vector. Infected RWPE-1 cells were selected with 0.5 μg/mL puromycin to obtain stable cell pool. For maintenance of the selection pressure, 0.25 μg/mL puromycin was added in the regular culture or cell assay.

### 4.7. Acinar Morphogenesis Assay

For regular monolayer cultures, RWPE-1 cells were incubated in K-SFM with 50 μg/mL bovine pituitary extract, 5 ng/mL human recombinant epidermal growth factor (rEGF) and 1% penicillin/streptomycin. To form acini, RWPE-1 cells were cultured according to the previous studies [19,64]. The cells (4 × 10^3^ for 8-well chamber slide and 8 × 10^3^ for 24-well culture plate) were seeded on culture plates coated with growth-factor-reduced Matrigel (356231, Corning Life Sciences, New York, NY, USA) (50 μL for eight-well chamber slide and 100 μL for 24-well culture plate) and incubated in assay medium (K-SFM with 5 ng/mL rEGF, 1% penicillin/streptomycin, 2% fetal bovine serum and 2% growth-factor-reduced Matrigel). The assay medium was refreshed every two days. For inducing gene expression, 0.2 μg/mL doxycycline was added in assay medium.

### 4.8. Western Analyses

In order to harvest the cells from Matrigel, acini were incubated in 0.25% Trypsin-EDTA at 37 °C for 10 min and then DMEM supplemented with 10% FBS was added to quench trypsin. The cells were washed once with PBS, centrifugated at 400× *g* for 5 min and suspended in a cell lysis buffer (50 mM Tris-HCl, pH7.5, 150 mM NaCl, 5 mM EDTA, 1% TritonX-100 and protease inhibitor cocktail). For harvest the cells from monolayer cultures (2D cultures), cells were collected in a cell lysis buffer by using cell scraper. After centrifugation at 14,000× *g* for 10 min at 4 °C, the supernatant was collected as whole cell lysate. Samples were separated by SDS-PAGE and transferred onto a PVDF membrane. The membrane was blocked by Gelatin-NET (50 mM Tris-HCl, pH 8.0, 0.25% gelatin, 150 mM NaCl, 5 mM EDTA and 0.05% Tween-20). The antibodies used in Western analyses include anti-CTEN (M3832, Spring Bioscience, Pleasanton, CA, USA), anti-GFP (SC-9996, Santa Cruz Biotechnology, Dallas, TX, USA), anti-p63 (ab735, Abcam, Cambridge, UK), anti-CK18 (MA5-12104, Invitrogen), anti-α-tubulin (T6074, Sigma-Aldrich), anti-p21 (2947, Cell Signaling Technology, Danvers, MA, USA), anti-p27 (2552, Cell Signaling Technology), anti-integrin β1 (GTX-128839, GeneTex, Irvine, CA, USA), anti-FAK (05-537, Millipore, Billerica, MA, USA), anti-FAK pY^397^ (700255, Invitrogen), peroxidase-labeled anti-mouse IgG (474-1806, KPL, Gaithersburg, MD, USA) and peroxidase-labeled anti-rabbit IgG (5220-0458, KPL). Western Lightning^®^ ECL Pro kit (PerkinElmer, Waltham, MA, USA) was used to detect peroxidase-labeled secondary antibodies and the signal was acquired by BioSpectrum Imaging System (UVP, Upland, CA, USA).

### 4.9. Immunofluorescence Microscopy

For following analyses by confocal microscopy, 3D cultures were carried out in eight-well chamber slides. Cells were fixed by 2% formaldehyde at room temperature and permeabilized by ice-cold 0.5% Triton-X100 at 4 °C. The cultures were washed with 100 mM glycine three times and then blocked by IF/BSA blocking buffer (1% BSA, 0.3% triton X-100 in PBS) containing 20 μg/mL goat anti-mouse F(ab’)_2_. Then the cultures were incubated with the primary antibody against CTEN (M3832, Spring Bioscience) diluted with IF/BSA blocking buffer at room temperature overnight and then washed with IF/BSA buffer three times. After incubation with the primary antibody, the cultures were incubated with the Alex Fluor 555-conjugated secondary antibody at room temperature for 1 h and then washed with IF/BSA buffer three times. For staining nucleic acid, the cultures were incubated with 2 μg/mL Hoechst 33,342 in PBS for 15 min and then washed with PBS. Images were acquired by using Zeiss LSM 780 (Zeiss, Thornwood, NY, USA).

### 4.10. Coimmunoprecipitation Assay

RWPE-1 cells were lysed in coimmunoprecipitation buffer (0.1% Triton X-100, 25 mM Tris-HCl, pH 8.0, 50 mM NaCl, 0.2 mM EDTA, 10 μg/mL aprotinin, 10 μg/mL leupeptin, 1 μg/mL pepstatine, and 1 μM PMSF). Cell lysates were then sheared by passing through a syringe needle, and the cell debris was removed by centrifugation at 14,000× *g* for 20 min at 4 °C. Five-hundred μg of the clarified cell lysates were incubated with 2 μg of an anti-CTEN antibody (M3832, Spring Bioscience) or nonspecific rabbit IgG (12-370, Millipore) by rotating at 4 °C for 4 h, followed by the addition of 50% of protein A-Sepharose slurry (17078001, GE Healthcare, Chicago, IL, USA) for 1 h. The protein A beads were collected by centrifugation and washed with coimmunoprecipitation buffer. Samples were then boiled in protein loading buffer and subjected to Western analyses.

### 4.11. GTP-RhoA Pull-Down Assay

The assays were performed using RhoA Pull-Down Activation Assay Biochem Kit (Cytoskeleton, Denver, CO, USA). Briefly, cells were washed with ice-cold PBS and lysed in Cell Lysis Buffer. Equivalent whole cell lysates were incubated with Rhotekin-RBD beads at 4 °C for 1 h. The beads were centrifuged at 4000× *g* at 4 °C for 1 min and washed with Wash Buffer. The bead samples were boiled in 2 × SDS sample buffer and then examined by Western analyses.

### 4.12. Statistical Analyses

Data were presented as mean ± standard deviation (SD). Student’s *t*-test was performed to determine statistical significance between two groups of data. The difference with value of *p* < 0.05 was considered as statistically significant.

## 5. Conclusions

CTEN is found enriched in basal subtypes of prostate epithelium and facilitates cell proliferation. Our study demonstrates that downregulation of CTEN is not only a shift during cell differentiation but also a requisite for normal prostate development. Moreover, during acinar formation, induction of CTEN expression reactivates FAK Y^397^ phosphorylation and disturbs acinar formation as well as luminal differentiation. This report provides novel insights into the physiological role of CTEN in prostate.

## Abbreviations

3D	Three dimensional
AR	Androgen receptor
BPH	Benign prostatic hyperplasia
CDK	Cyclin-dependent kinase
CK18	Cytokeratin 18
CK5	Cytokeratin 5
CK8	Cytokeratin 8
CTEN	C-terminal tensin-like protein
EGFP	Enhanced green fluorescent protein
ERK	extracellular-signal regulated kinase
FAK	Focal adhesion kinase
GEO	Gene Expression Omnibus
MAPK	mitogen-activated protein kinase
PrEC	Prostate epithelial cell
PrSC	Prostate stromal cell
PrSMC	Prostate smooth muscle cell
PSA	Prostate-specific antigen
PSCA	Prostate stem cell antigen
PTB	Phosphotyrosine-binding
SH2	Src homology 2
rEGF	Recombinant epidermal growth factor
